# Detecting anisotropic segmental dynamics in disordered proteins by cross-correlated spin relaxation

**DOI:** 10.5194/mr-2-557-2021

**Published:** 2021-07-06

**Authors:** Clemens Kauffmann, Irene Ceccolini, Georg Kontaxis, Robert Konrat

**Affiliations:** 1 Department of Structural and Computational Biology, Max Perutz Laboratories, University of Vienna, Campus-Vienna-Biocenter 5, 1030 Vienna, Austria

## Abstract

Among the numerous contributions of Geoffrey Bodenhausen to NMR spectroscopy, his developments in the field of spin-relaxation methodology and theory will definitely have a long lasting impact. Starting with his seminal contributions to the excitation of multiple-quantum coherences, he and his group thoroughly investigated the intricate relaxation properties of these “forbidden fruits” and developed experimental techniques to reveal the relevance of previously largely ignored cross-correlated relaxation (CCR) effects, as “the essential is invisible to the eyes”. Here we consider CCR within the challenging context of intrinsically disordered proteins (IDPs) and emphasize its potential and relevance for the studies of structural dynamics of IDPs in the future years to come. Conventionally, dynamics of globularly folded proteins are modeled and understood as deviations from otherwise rigid structures tumbling in solution. However, with increasing protein flexibility, as observed for IDPs, this apparent dichotomy between structure and dynamics becomes blurred. Although complex dynamics and ensemble averaging might impair the extraction of mechanistic details even further, spin relaxation uniquely encodes a protein's structural memory. Due to significant methodological developments, such as high-dimensional non-uniform sampling techniques, spin relaxation in IDPs can now be monitored in unprecedented resolution. Not embedded within a rigid globular fold, conventional ${}^{15}N$ spin probes might not suffice to capture the inherently local nature of IDP dynamics. To better describe and understand possible segmental motions of IDPs, we propose an experimental approach to detect the signature of anisotropic segmental dynamics by quantifying cross-correlated spin relaxation of individual ${}^{15}N^{1}H^{N}$ and ${}^{13}{C^{\prime }}^{13}C^{\alpha }$ spin pairs. By adapting Geoffrey Bodenhausen's symmetrical reconversion principle to obtain zero frequency spectral density values, we can define and demonstrate more sensitive means to characterize anisotropic dynamics in IDPs.

## 1 Introduction

Geoffrey Bodenhausen's 70th anniversary marks an ideal occasion to take a fresh look at some of his numerous contributions to spin-relaxation methodology and theory. By considering his experiments within the challenging context of intrinsically disordered proteins (IDPs), we want to emphasize their potential and relevance in the future years to come. Arguably, this rediscovery might require some collective effort, as current trends appear to point in the opposite direction. As Paul Schanda put it recently: “The popularity of detailed spin-relaxation measurements in liquids, *en vogue* 10 or 20 years ago, is declining; [ … ] with lengthy measurements it is not easy to gain much more insight than `loops are more flexible than secondary structures', which often does not answer mechanistic questions.” p. 3–4. While intentionally exaggerated, this statement does point to some of the inherent limitations of relaxation experiments. Owing to their convoluted nature, spin relaxation reports on protein dynamics only in ambiguous terms. A variety of stochastic processes can lead to time correlation functions (TCFs) of identical shape and form p. 1–7. In addition, the TCF is not probed directly, only its spectral density; i.e., its Fourier transform is sampled at a few select frequencies. Thus, with far more detailed structural models at hand, protein dynamics are often understood as mere perturbations of otherwise rigid bodies tumbling in solution [Bibr bib1.bibx36]. Relaxation experiments commonly employed to calculate protein structures, such as nuclear Overhauser effects (NOEs) and paramagnetic relaxation enhancements (PREs), are usually modeled without accounting for their dynamic nature [Bibr bib1.bibx26]. In a sense, protein dynamics might appear separate from protein structure, at least within the structure–function paradigm.

However, with increasing protein flexibility this apparent dichotomy becomes blurred as structure and dynamics can no longer be considered independent of each other. While complex dynamics and ensemble averaging obfuscate mechanistic details even further, the structural information content of relaxation parameters becomes increasingly apparent. In comparison to simple population-averaged quantities, such as chemical shifts or scalar couplings, spin relaxation uniquely encodes a system's structural memory, i.e., the temporal persistence of concerted motions and structural arrangements. Somewhat counterintuitively, spin-relaxation experiments are among the prime sources of structural information available for disordered systems. However, due to a general lack of analytical descriptions for IDP dynamics [Bibr bib1.bibx43], this notion has been of somewhat academic nature until the recent past. Continuous developments in molecular dynamics (MD) simulation protocols [Bibr bib1.bibx48] demonstrate how this gap can finally be bridged, allowing us to validate, refine and/or analyze dynamic ensemble representations of proteins in solution [Bibr bib1.bibx29]. With the necessary timescales becoming increasingly accessible [Bibr bib1.bibx61] and the spectral resolution provided by high-dimensional non-uniform sampling experiments to overcome the problem of spectral overlap [Bibr bib1.bibx21], spin relaxation in IDPs can be investigated in unprecedented fashion.

This aspect alone suggests a systematic reassessment and evaluation of less commonly employed experiments. Far more pressing, in our opinion, is the inherently local nature of spin relaxation in IDPs. In contrast to folded proteins, spins in IDPs are not embedded within a fixed molecular tumbling frame. Thus, a single ${}^{15}N$ nucleus per residue as a dynamic probe might not suffice to capture the underlying motions in adequate detail. While detecting and quantifying the presence of anisotropy in IDP dynamics might seem like a rather academic endeavor, it represents an important stepping stone towards the structural interpretation of other experiments. As we recently demonstrated, an appropriate estimate for the average correlation time is an important prerequisite for the angular evaluation of cross-correlated relaxation (CCR) of remote spins [Bibr bib1.bibx30].

More immediate in its structural implications would be the presence of diffusion anisotropy, which has been hypothesized to be of substantial size even in highly disordered proteins such as α -synuclein. Specifically, segmental tumbling of α -helical and extended chain conformations has been implied to lead to pronounced diffusion anisotropy effects for intraresidual and sequential ${}^{1}H{-}^{1}H$ NOEs [Bibr bib1.bibx72]. At the same time, the 3D GAF model [Bibr bib1.bibx4] has been invoked to further rationalize the presence of anisotropic dynamics on the local scale of the peptide plane. This model has recently been reframed by [Bibr bib1.bibx55] to analyze MD-simulated ${}^{15}N$ relaxation of a partially disordered protein. In essence, it was demonstrated that ${\mathrm{NH}}^{N}$ TCFs are well described by the $C^{\alpha }C^{\alpha }$ TCFs of the same peptide plane as long as variations of the flanking dihedral angles and ${\mathrm{NH}}^{N}$ librations are accounted for. Explicit corrections for possible effects of anisotropic segmental tumbling were not required. However, noticeable deviations could be observed for the transverse ${}^{15}N$ relaxation of the slowly moving residual α -helix. [Bibr bib1.bibx40] have reported pronounced diffusion anisotropy within the α -helical region of an otherwise disordered construct. Flexible residues were affected noticeably less. It was suggested this might be due to their average orientation in the molecular tumbling frame [Bibr bib1.bibx40]. The SRLS model of [Bibr bib1.bibx64] and [Bibr bib1.bibx42] also predicts pronounced anisotropy for α -helices and β -sheets. However, loops and terminal chain segments appear isotropic, asserting that proteins with substantial internal mobility are best represented by an isotropic global diffusion tensor [Bibr bib1.bibx73].

Arguably, this somewhat ambiguous body of evidence illustrates the inevitable difficulties that come with extending concepts of folded proteins to IDPs. In fact, many of the above observations might very well be case-dependent. In the present study, we want to approach this question in a more agnostic manner. Are there experimental ways to better detect the signature of anisotropic dynamics in IDPs? At what level of evidence could we evoke the mental image of extended chains and α -helical segments tumbling in solution? The principal difficulty in characterizing these structural elements lies in their translational periodicity. In an α -helix, ${\mathrm{NH}}^{N}$ vectors are strongly aligned along the main axis, while in an extended chain they are oriented perpendicularly. In order to detect possible orientational biases in their relaxation behavior, additional spin probes with different orientations must be considered. While $C^{\alpha }H^{\alpha }$ might be suitable for α -helices [Bibr bib1.bibx3], its orientation is too similar to ${\mathrm{NH}}^{N}$ in the extended chain conformation. Moreover, since it does not share a peptide plane with ${\mathrm{NH}}^{N}$ , it varies as a function of ϕ and ψ as do the ${}^{1}H{-}^{1}H$ intraresidual and sequential NOEs. Spin probes within the same peptide plane and thus less ambiguous orientations would certainly be preferable.

For IDPs in particular, [Bibr bib1.bibx27] have shown that the ${\mathrm{NH}}^{N}$ spectral density is best mapped by a combination of transversal and longitudinal CCR rates, employing Geoffrey Bodenhausen's symmetrical reconversion principle [Bibr bib1.bibx46]. Together with Bodenhausen and coworkers, this concept was later extended to measure the zero frequency spectral density in a single experiment [Bibr bib1.bibx28]. By translating these concepts to the $C^{\prime }C^{\alpha }$ spin pair, we want to derive and demonstrate more sensitive means to detect anisotropic segmental dynamics in IDPs.

## 2 Theory

Our aim is to define an experimental measure for anisotropic segmental dynamics in IDPs. While the measure itself should be general, the considered source of anisotropy will be rather specific. To assess the sensitivity of the proposed protocol, we resort to the simplified image of extended chain and α -helical segments tumbling in solution as previously asserted by [Bibr bib1.bibx38]. Before considering experimental aspects, we start by defining the spectral density. Sampled at zero frequency and/or (combinations of) the involved Larmor frequencies, it constitutes the fundamental quantity of all spin-relaxation experiments:

1 $$J_{u,v}(\omega )=\int_{0}^{\infty }C_{u,v}(t)\mathrm{cos}(\omega t)\;dt$$

with the time correlation function (TCF),

2 $$C_{u,v}(t)=\langle P_{2}(u(0)\cdot v(t))\rangle ,$$

where $P_{2}(x)=1.5x^{2}-0.5$ is the second-order Legendre polynomial, u and v represent either dipolar unit vectors or principal components of chemical shift anisotropy (CSA) tensors. Note that our simplified definition of the TCF implicitly assumes that time-dependent distance fluctuations factorize and can thus be absorbed into constant coefficients. This requirement will be well satisfied for the spins considered henceforth.

For most processes, the TCF can be described as a sum or distribution of exponential decays [Bibr bib1.bibx36]:

3 $$C_{u,v}(t)=\sum_{k=0}^{N}a_{k}e^{-t/\tau_{k}}.$$

Evaluating at t=0 yields a type of normalization condition,

4 $$\sum_{k=0}^{N}a_{k}=C_{u,v}(0)=\langle P_{2}(u(0)\cdot v(0))\rangle ,$$

which equates to 1 for the familiar case of auto-correlation ( u=v ). For cross-correlation ( u≠v ), Eq. ([Disp-formula Ch1.E4]) is bounded within [-0.5,1] .

The spectral density of Eq. ([Disp-formula Ch1.E3]) is a sum of Lorentzian functions

5 $$J_{u,v}(\omega )=\sum_{k=0}^{N}a_{k}\frac{\tau_{k}}{1+(\omega \tau_{k}{)}^{2}}$$

Note that, depending on how the TCF and the spectral density are defined, Eq. ([Disp-formula Ch1.E5]) might come with additional coefficients such as the familiar factor of $\frac{2}{5}$ [Bibr bib1.bibx36]. We prefer the above definitions as they highlight $J_{u,v}(\omega )$ as a weighted average. At zero frequency all $\tau_{k}$ 's are weighted equally; i.e., J(0) encodes the average correlation time. With increased frequency, the impact of larger $\tau_{k}$ becomes less pronounced. This is illustrated in Fig. [Fig Ch1.F1] for a selection of Larmor frequencies assuming a magnetic field strength of 18.8 T (800 MHz proton Larmor frequency).

**Figure 1 Ch1.F1:**
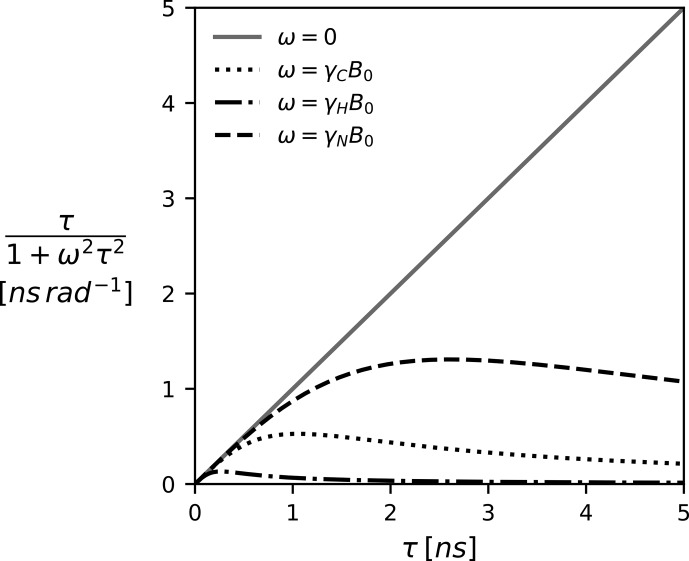
The Lorentzian as a function of the correlation time τ and the (Larmor) frequency. The spectral density J(ω) , which is modeled as a linear combination of Lorentzian functions, can be pictured as a weighted average of all correlation times τ . Since J(0) weights all correlation times equally, it represents the component most sensitive to correlation times >1 ns. The magnetic field $B_{0}$ is 18.8 T (800 MHz proton Larmor frequency).

Detecting anisotropy amounts to quantifying orientational biases in $a_{k}$ and $\tau_{k}$ . Similarly to [Bibr bib1.bibx38], we will attribute these biases to relative orientations in idealized extended chain and α -helical segments. These structural elements are well described by an axially symmetric diffusion tensor, which yields the following expression for the spectral density [Bibr bib1.bibx63]:

6 $$J_{u,v}(\omega )=\sum_{k=0}^{2}A_{k}(u,v)\frac{\tau_{k}}{1+(\omega \tau_{k}{)}^{2}},$$

where

a0≡A0(u,v)=P2(θu)P2(θv),a1≡A1(u,v)=0.75sin⁡(2θu)sin⁡(2θv)cos⁡(ϕu-ϕv),7a2≡A2(u,v)=0.75sin⁡2(θu)sin⁡2(θv)cos⁡(2ϕu-2ϕv),

and θ and ϕ denote the polar angles in the tumbling frame. The $\tau_{k}$ 's correspond to the inverted eigenvalues of the axially symmetric diffusion tensor:

τk=6D⟂+k2D∥-D⟂-18=D⟂-16+k2D∥D⟂-1-1

with k=0,1,2 . With u and v alone, Eq. ([Disp-formula Ch1.E6]) would not allow us to distinguish between size effects in $\tau_{k}$ (i.e., “segment length”) and orientational biases in $A_{k}(u,v)$ (i.e., “secondary structure”). To quantify the anisotropy ( $\frac{D_{∥}}{D_{⟂}}$ ) only, we consider another interaction described by a second set of vectors ( x , y ) embedded with different orientations in the same tumbling frame and focus our attention on J(0) for three reasons. First and foremost, J(0) is the component most sensitive to the $\tau_{k}\geq 1$ ns (cf. Fig. [Fig Ch1.F1]) commonly associated with tumbling motions [Bibr bib1.bibx29]. Secondly, the zero frequency does not depend on the type of nuclei involved. Lastly, J(0) allows us to define a convenient ratio,

Ju,v(0)Jx,y(0)=D⟂-1∑k=02Ak(u,v)(6+k2(D∥D⟂-1))-1D⟂-1∑k=02Ak(x,y)(6+k2(D∥D⟂-1))-19=∑k=02Ak(u,v)(6+k2(D∥D⟂-1))-1∑k=02Ak(x,y)(6+k2(D∥D⟂-1))-1

such that the explicit size dependency cancels out. In general, if ( u , v ) and ( x , y ) have fixed relative orientations and experience the same dynamics, we obtain the isotropic edge case:

10 $$\frac{J_{u,v}(0)}{J_{x,y}(0)}=\frac{P_{2}(u\cdot v)}{P_{2}(x\cdot y)}.$$

Thus, the ratio (Eq. 9) encodes a simple and intuitive relation: isotropic motions tend towards the limit (Eq. [Disp-formula Ch1.E10]), while anisotropic motions deviate from it. Importantly, the source of anisotropy is not essential. The invoked model of a tumbling symmetric top, Eq. ([Disp-formula Ch1.E6]), does not need to apply in any strict sense; rather, it is used to assess the sensitivity of the J(0) ratio (Eq. 9) to its associated features. Presuming that segmental arrangements are sufficiently stable, remnants of Eq. ([Disp-formula Ch1.E6]) might still be detectable even in the case of pronounced local mobility.

In general, assessing the effects of internal motions on Eq. ([Disp-formula Ch1.E6]) is not straightforward. Even for folded proteins, the commonly employed model-free (MF) [Bibr bib1.bibx36] and extended MF [Bibr bib1.bibx10] approaches are not strictly applicable in the case of anisotropic diffusion [Bibr bib1.bibx13]. For CCR in particular, the proposed adaptations can become quite intricate [Bibr bib1.bibx65]. Geoffrey Bodenhausen and coworkers have investigated this topic in a series of publications [Bibr bib1.bibx16]. Given the toy nature of the considered model, we will assess the presence of fast, isotropic motions as simply as possible by introducing a fourth Lorentzian:

Ju,v(ω)=S2∑k=02Ak(u,v)τk1+(ωτk)211+(1-S2)P2(u⋅v)τ31+(ωτ3)2

with $\tau_{3}^{-1}=\tau_{\text{int}}^{-1}+4D_{⟂}+2D_{∥}$ , where $\tau_{\text{int}}$ is the average correlation time of the fast internal motion. The generalized order parameter $S^{2}\in [0,1]$ acts as a weight balancing the contributions of slow anisotropic tumbling and fast isotropic motions. To account for the angular relation between u and v , $a_{3}$ necessarily corresponds to $P_{2}(u\cdot v)$ , which follows intuitively from condition Eq. ([Disp-formula Ch1.E4]), assuming a fixed angle between u and v .

Of course, the additional Lorentzian can be rationalized in terms of established models. As shown in the Appendix, Eq. (11) corresponds to a simplification of the MF-like extension proposed by [Bibr bib1.bibx19], Eq. (A1) and generalized by [Bibr bib1.bibx67] and [Bibr bib1.bibx65]. In this model, the generalized order parameters depend on k as well; i.e., the weighting between isotropic and anisotropic contributions can indeed vary between different k 's. Using a single order parameter is exact only if the molecule is fully rigid ( $S^{2}=1$ ) or if the dynamics are entirely isotropic ( $\frac{D_{∥}}{D_{⟂}}=1$ or $S^{2}=0$ ). In the case of pronounced diffusion anisotropy and intermediate local mobility, Eq. (11) is only an approximate interpolation between these edge cases. Still, it is worth noting that the use of a single order parameter is both a common heuristic to account for the effects of local dynamics on experimental CCR rates [Bibr bib1.bibx67] and an established approximation for sufficiently small angles between u and v [Bibr bib1.bibx63]. To keep the amount of parameters manageable, possible differences in local dynamics for different k and/or between ( u , v ) and ( x , y ) are not reflected in Eq. (11). Systematic differences in local peptide plane dynamics [Bibr bib1.bibx7] will necessarily result in deviations from the isotropic case, Eq. ([Disp-formula Ch1.E10]). While local motions could be modeled in more detail to better match the shape of the TCF using, for example, an extended MF approach [Bibr bib1.bibx10], correlation time distributions [Bibr bib1.bibx24] or dynamic detectors [Bibr bib1.bibx59], we only intend to divide J(0) , i.e., the TCF's enclosed area, into contributions with and without orientational biases in an intuitive and simple manner. Attributed solely to the $A_{k}$ , Eq. (7), we now consider the effect of these biases from an experimental perspective.

## 3 Methods

We will assume the canonical peptide plane geometry of [Bibr bib1.bibx11] as depicted in Fig. [Fig Ch1.F2], including approximate principal components of the CSA tensors for ${}^{15}N$ and ${}^{13}C^{\prime }$ adapted from Geoffrey Bodenhausen and coworkers [Bibr bib1.bibx8].

**Figure 2 Ch1.F2:**
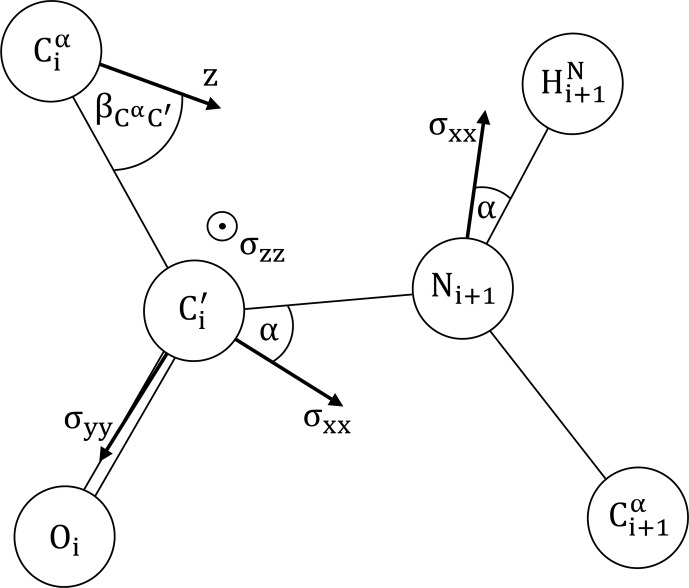
The peptide plane as defined by [Bibr bib1.bibx11]: $C^{\alpha }-C^{\prime }=1.53$ Å, $C^{\prime }-O=1.24$ Å, $C^{\prime }-N=1.32$ Å, $N-C^{\alpha }=1.47$ Å. $C^{\alpha }-C^{\prime }-O=121$ ${}^{\circ }$ , $C^{\alpha }-C^{\prime }-N=114$ ${}^{\circ }$ , $O-C^{\prime }-N=125$ ${}^{\circ }$ , $C^{\prime }-N-H=123$ ${}^{\circ }$ , $C^{\prime }-N-C^{\alpha }=123$ ${}^{\circ }$ and $H-N-C^{\alpha }=114$ ${}^{\circ }$ . N-H=1.04Å is taken from [Bibr bib1.bibx45]. The ${}^{15}N$ and ${}^{13}C^{\prime }$ CSA principal components are adapted from Bodenhausen and coworkers [Bibr bib1.bibx8]. ${}^{15}N$ : $\Delta_{N}\approx \sigma_{xx}-\sigma_{yy}\approx \sigma_{xx}-\sigma_{zz}=170$ ppm, α=20 ${}^{\circ }$ . ${}^{13}C^{\prime }$ : $\sigma_{xx}=249.4$ ppm, $\sigma_{zz}=87.9$ ppm, α=37 ${}^{\circ }$ . $\sigma_{yy}=191.1$ ppm is obtained from the average chemical shift of ubiquitin (BMRB 17769) [Bibr bib1.bibx12], following the suggested calibration [Bibr bib1.bibx8]. The main axis z of the diffusion anisotropy tensor is assumed to lie in the peptide plane. Its orientation is encoded by $\beta_{C^{\alpha }C^{\prime }}$ .

As demonstrated by [Bibr bib1.bibx27], the spectral densities of IDPs are best mapped by combining the transversal ( $\Gamma_{xy}$ ) and the longitudinal ( $\Gamma_{z}$ ) CCR rates between the ${}^{15}N$ CSA and the ${\mathrm{NH}}^{N}$ dipole. Employing the expressions of Bodenhausen and coworkers [Bibr bib1.bibx8], we have

12ΓxyN/NH=kN/NHΔN4JNH,xx(0)+3JNH,xx(ωN),13ΓzN/NH=kN/NHΔN6JNH,xx(ωN)kN/NH=25124πμ0γnγhrNH3B0γn,

where $\mu_{0}$ is the vacuum permeability, 
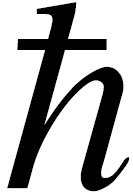
 is the reduced Planck constant, γ is the gyromagnetic ratio, r is the distance between the nuclei, $B_{0}$ is the magnetic field strength and $\Delta_{N}=(\sigma_{xx}-\sigma_{yy})=(\sigma_{xx}-\sigma_{zz})$ is the size difference of the ${}^{15}N$ CSA principal components (in ppm). Mapping $J_{\mathrm{NH},xx}(0)$ amounts to the simple subtraction $\Gamma_{xy}^{N/\mathrm{NH}}-0.5\Gamma_{z}^{N/\mathrm{NH}}$ .

To complement these rates, we consider their counterparts for the ${}^{13}C^{\prime }$ CSA and the $C^{\prime }C^{\alpha }$ dipole:

ΓxyC′/C′Cα=kC′/C′Cα[(σxx-σzz)⋅4JC′Cα,xx(0)+JC′Cα,xx(ωC)+(σyy-σzz)14⋅4JC′Cα,yy(0)+3JC′Cα,yy(ωC)],ΓzC′/C′Cα=kC′/C′Cα[(σyy-σzz)6JC′Cα,xx(ωC)15+(σyy-σzz)6JC′Cα,yy(ωC)],kC′/C′Cα=25124πμ0γc2rC′Cα3B0γc

with xx , yy and zz referring to the principal components of the fully anisotropic ${}^{13}C^{\prime }$ CSA tensor. Again, high frequency contributions can be eliminated via the linear combination $\Gamma_{xy}^{C^{\prime }/C^{\prime }C^{\alpha }}-0.5\Gamma_{z}^{C^{\prime }/C^{\prime }C^{\alpha }}$ .

While the measurement of transverse CCR is well established, longitudinal CCR has been studied considerably less. In part this is due to subtleties of the involved relaxation pathways which involve multi-exponential auto- and cross-correlated relaxation effects. Another reason lies in technical limitations as longitudinal relaxation rates are generally smaller due to their Larmor frequency dependence. Notably, this effect is far less pronounced for the smaller correlation times present in IDPs (cf. Fig. [Fig Ch1.F1]). While ${}^{15}N^{1}H^{N}$ relaxation is well understood and several sensitive NMR techniques have been proposed to measure $\Gamma_{xy}^{N/\mathrm{NH}}$ and $\Gamma_{z}^{N/\mathrm{NH}}$ [Bibr bib1.bibx63], ${}^{13}C^{\prime }$ relaxation is generally more problematic [Bibr bib1.bibx14]. Since we could not find any previous attempts to measure the longitudinal CCR rate $\Gamma_{z}^{C^{\prime }/C^{\prime }C^{\alpha }}$ in the literature, we see fit to assess its general feasibility.

Aside from the symmetrical reconversion principle of Bodenhausen and coworkers [Bibr bib1.bibx46], ${}^{13}C^{\prime }{/}^{13}{C^{\prime }}^{13}C^{\alpha }$ CSA-DD CCR can be measured either by monitoring the relaxation asymmetry of the ${}^{13}{C^{\prime }}^{13}C^{\alpha }$ doublet or by means of a “quantitative gamma” experiment in which the sum and difference of the ${}^{13}C^{\prime }$ doublet relaxation are measured independently. In contrast to previous approaches relying on two separate experiments (“reference” and “cross”) [Bibr bib1.bibx57], we determine $\Gamma_{z}^{C^{\prime }/C^{\prime }C^{\alpha }}$ by quantifying the different longitudinal relaxation in the ${}^{13}{C^{\prime }}^{13}C^{\alpha }$ doublet recorded in a non-constant-time ${}^{13}C^{\prime }$ evolution following the relaxation period. Transverse relaxation $\Gamma_{xy}^{C^{\prime }/C^{\prime }C^{\alpha }}$ is measured by more conventional quantification of differential line broadening of the ${}^{13}{C^{\prime }}^{13}C^{\alpha }$ doublet recorded in constant-time mode.

To obtain sufficient spectral resolution, the CCR rates are measured directly from the intensity difference in a ${}^{13}C^{\alpha }$ -coupled 3D HNCO experiment: (i) in the case of transversal CCR by quantification of differential line broadening of the ${}^{13}{C^{\prime }}^{13}C^{\alpha }$ doublet during constant-time ${}^{13}C^{\prime }$ evolution and (ii) for longitudinal CCR during real-time ${}^{13}C^{\alpha }$ -coupled ${}^{13}C^{\prime }$ evolution preceded by a longitudinal relaxation delay T during which ${}^{13}C^{\prime }{/}^{13}{C^{\prime }}^{13}C^{\alpha }$ CSA-DD CCR is active. This approach yields reliable longitudinal CCR rates as long as the mixing time T is short compared to ${}^{13}C^{\prime }$ $T_{1}$ relaxation. Typical data obtained for the small globular protein ubiquitin are shown in Fig. [Fig Ch1.F3]

**Figure 3 Ch1.F3:**
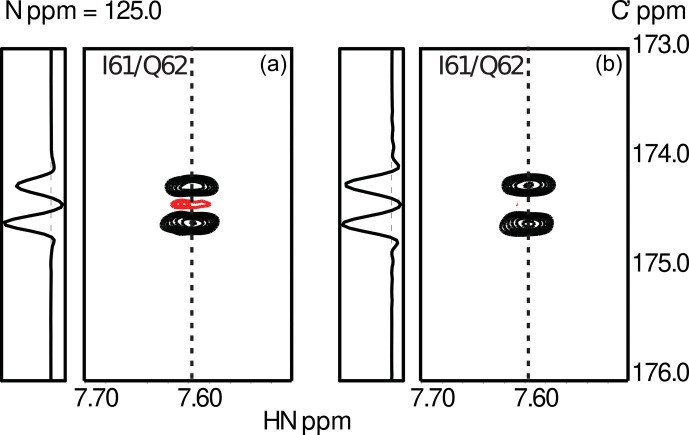
Experimental results from the measurements of transverse **(a)** and longitudinal **(b)** ${}^{13}C^{\prime }{/}^{13}{C^{\prime }}^{13}C^{\alpha }$ CSA-DD CCR as described in Sect. [Sec Ch1.S3]. Data were obtained using ubiquitin, a small globular protein of 76 residues. The figure shows the spectral region for the peptide plane spanning residues I61/Q62. The asymmetry of the ${}^{13}C^{\alpha }$ doublet is visible in the cross-sections taken at the positions indicated by dashed lines. As expected, CCR effects are more pronounced in the case of transverse relaxation.

To suppress ${}^{13}{C^{\prime }}^{13}C^{\alpha }$ cross-relaxation, a ${}^{13}C^{\prime }$ is inverted in the middle of the relaxation delay T . Additional unwanted CCR pathways involving the ${}^{13}C^{\prime }$ CSA and ${}^{13}{C^{\prime }}^{1}H{/}^{13}{C^{\prime }}^{15}N$ dipoles are suppressed by ${}^{1}H$ decoupling and ${}^{15}N$ inversion. As the ${}^{2}J$ couplings to remote carbons ( ${}^{13}{C^{\prime }}^{13}C^{\beta }$ and ${}^{13}C_{i}^{\prime }$ ${}^{13}C_{i+1}^{\alpha }$ ) are not resolved, CCR effects can be expected to average out for short and intermediate mixing times. While up to 20 % in size of the ${}^{13}{C^{\prime }}^{13}C^{\alpha }$ CCR, the $\pm {}^{13}{C^{\prime }}^{13}C^{\beta }$ CCR components contribute to the same line. The CCR rates are obtained from the ${}^{13}{C^{\prime }}^{13}C^{\alpha }$ doublet as $\mathrm{log}(\frac{I_{a}}{I_{b}})/2T$ . Details of the pulse sequence and NMR parameters will be given elsewhere. Two exemplary ${}^{13}{C^{\prime }}^{13}C^{\alpha }$ doublets measured for I61/Q62 in human ubiquitin are shown in Fig. [Fig Ch1.F3].

With the general feasibility of the measurements demonstrated, we can now define a ratio Q analogous to Sect. [Sec Ch1.S2], Eq. (9):

Q≡ΓxyC′/C′Cα-0.5ΓzC′/C′CαΓxyN/NH-0.5ΓzN/NH16=4kC′/C′Cα(σxx-σzz)JC′Cα,xx(0)+(σyy-σzz)JC′Cα,yy(0)4kN/NHΔNJNH,xx(0).

To assess the sensitivity of Q (Eq. 16), it is evaluated according to Eqs. (7), (8), (11), (12)–(15) with $\tau_{3}^{-1}=\tau_{\text{int}}^{-1}+4D_{⟂}+2D_{∥}=\tau_{\text{int}}^{-1}+\tau_{\text{eff}}^{-1}$ under the following conditions: as specified in Fig. [Fig Ch1.F2], all CSA tensors have fixed orientation and size. Following [Bibr bib1.bibx38], the main axis, z , of the axially symmetric diffusion tensor is assumed to lie in the peptide plane; hence, Q is a function of the polar angles θ only; see Eq. (7). Defining the $C^{\alpha }C^{\prime }$ orientation as 0 ${}^{\circ }$ reference, the main axis is rotated from 0 to 180 ${}^{\circ }$ towards the ${\mathrm{NH}}^{N}$ vector assuming anisotropy values $\frac{D_{∥}}{D_{⟂}}$ of 1.5 and 2.5, effective tumbling times $\tau_{\text{eff}}=(4D_{⟂}+2D_{∥}{)}^{-1}$ of 1 and 2.5 ns, internal correlation times $\tau_{\text{int}}$ of 100 and 500 ps and order parameters $S^{2}$ between 0 an 1. The magnetic field strength $B_{0}$ is the same for all rates and thus cancels out. The results are summarized in Fig. [Fig Ch1.F4].

**Figure 4 Ch1.F4:**
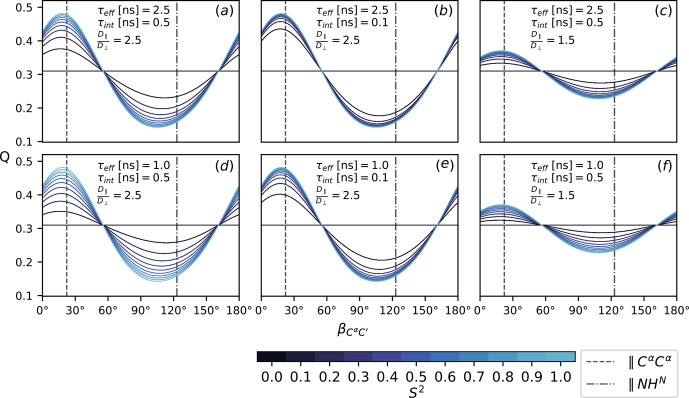
The ratio Q , Eq. (16), as a function of the diffusion tensor orientation denoted by $\beta_{C^{\alpha }C^{\prime }}$ , Fig. [Fig Ch1.F2]. Dashed lines indicate the orientation of the ${\mathrm{NH}}^{N}$ and the $C^{\alpha }C^{\alpha }$ vector. All rates are calculated according to Eqs. (7), (8), (11), (12)–(15) with $\tau_{3}^{-1}=\tau_{\text{int}}^{-1}+\tau_{\text{eff}}^{-1}$ . Order parameters from 0 and 1 are color-coded. Panels **(a–f)** show Q for different choices of $\tau_{\text{eff}}$ , $\tau_{\text{int}}$ and $\frac{D_{∥}}{D_{⟂}}$ . The magnetic field strength, $B_{0}$ , is the same for all rates.

## 4 Results and discussion

Experimental considerations necessarily result in compromises. The fully anisotropic ${}^{13}C^{\prime }$ CSA not only leads to spectral density contributions of two perpendicular components, it is also subject to considerable variations [Bibr bib1.bibx41]. One might be tempted to avoid the uncertainties and complications that come with the ${}^{13}C^{\prime }$ CSA by considering dipolar relaxation only. However, compared to the ${\mathrm{NH}}^{N}$ spin pair, the small gyromagnetic ratios and long internuclear distances of other dipoles lead to far smaller and less sensitive rates [Bibr bib1.bibx6]. In addition, the J(0) components are generally neither dominant nor easily separated. The ${}^{13}C^{\prime }$ CSA both provides effective means of relaxation and allows for a straightforward extraction of J(0) components. With an approximate ratio $(\sigma_{xx}-\sigma_{zz})/(\sigma_{yy}-\sigma_{zz})\approx 1.5$ and the beneficial orientation of the $C^{\prime }C^{\alpha }$ vector, the $J_{C^{\prime }C^{\alpha },xx}(\omega )$ contribution is generally far more pronounced: for $30{}^{\circ }\leq \alpha \leq 44{}^{\circ }$ , the TCF amplitudes would be $0.48\leq P_{2}(C^{\prime }C^{\alpha }\cdot xx)\leq 0.79$ and $0.02\geq P_{2}(C^{\prime }C^{\alpha }\cdot yy)\geq -0.29$ based on the geometry of Fig. [Fig Ch1.F2].

Figure [Fig Ch1.F4] shows the ratio Q (Eq. 16) for different choices of $\tau_{\text{eff}}$ , $\tau_{\text{int}}$ , $S^{2}$ and $\frac{D_{∥}}{D_{⟂}}$ as a function of the diffusion tensor orientation. The main axis is assumed to lie in the peptide plane with the orientation denoted relative to $C^{\prime }C^{\alpha }$ in terms of the angle $\beta_{C^{\prime }C^{\alpha }}$ ; see Fig. [Fig Ch1.F2]. Comparing all panels (a)–(f) at once, it can be seen that the isotropic ( $S^{2}=0$ ) baseline at around 0.3 is independent of the specific correlation times $\tau_{\text{eff}}$ and $\tau_{\text{int}}$ ; see Eq. ([Disp-formula Ch1.E10]). The same value is obtained for $\frac{D_{∥}}{D_{⟂}}=1$ , which is easily assessed from the convergence behavior for different anisotropy values in (c) and (f) and (a), (b), (d) and (e). How strongly Q reports on the asserted presence of diffusion anisotropy depends on the $S^{2}$ -mediated weight difference between the orientation-dependent $A_{k}\tau_{k}$ (7) and the isotropic $\tau_{\text{eff}}$ . Both higher overall tumbling $\tau_{\text{eff}}$ and smaller isotropic motions $\tau_{\text{int}}$ yield a more sensitive Q for increasingly smaller order parameters $S^{2}$ ; see panels (a), (b), (d) and (e). Of course, this interpolation is only approximate and can become more intricate if the local motions are considered in more detail. In this simplified case, the particular choice of $\tau_{\text{int}}$ and $S^{2}$ is to some extent arbitrary as $A_{k}\tau_{k}$ , $\tau_{\text{int}}$ and $S^{2}$ merely weight the isotropic and anisotropic contributions to the TCF's enclosed area J(0) . Still, the range $\tau_{\text{eff}}\geq 1\;\mathrm{ns}>\tau_{\text{int}}$ was chosen based on timescales recently reported for MD simulations of IDPs [Bibr bib1.bibx29].

Besides the obvious influence of $\frac{D_{∥}}{D_{⟂}}$ itself, Q strongly depends on the orientation of the diffusion tensor. The orientations of the ${\mathrm{NH}}^{N}$ and the $C^{\alpha }C^{\alpha }$ vectors are highlighted in all panels (a)–(f). In an extended chain, $C^{\alpha }C^{\alpha }$ is approximately parallel to the main axis, while ${\mathrm{NH}}^{N}$ stands perpendicular to it or vice versa for an α -helix. Both orientations correspond well to the minimum and maximum of Q . The range of Q depends on the size of the anisotropy $\frac{D_{∥}}{D_{⟂}}$ . For a value of 2.5, as was previously asserted for α -synuclein [Bibr bib1.bibx38], the effect on Q can be quite substantial (panels a, b, d, e). For $\frac{D_{∥}}{D_{⟂}}=1.5$ , it is far less pronounced (panels c and f).

We conclude that, if the concept of anisotropic tumbling of segmental α -helices and extended chains is reasonably applicable and sufficiently pronounced, Q would allow us to detect its signature. Actual quantification of $\frac{D_{∥}}{D_{⟂}}$ is of course obstructed by the limited validity of the asserted dynamic model. While the presence of relaxation-active tumbling motions does imply a certain degree of local rigidity, the structural heterogeneity of IDPs certainly challenges many of the simplifying assumptions made. Still, the ratio Q might give an indication of how relevant these concepts are for different protein systems, e.g., a partially folded region [Bibr bib1.bibx55] or a highly disordered segment [Bibr bib1.bibx38]. While particularly sensitive to large correlation times, Q will report on all sources of anisotropy present in J(0) . Differences in local mobility, CSA tensor variations, overall structural flexibility and experimental uncertainties will certainly shift and blur the ratio expected for isotropic motions. Still, if we assume a set of consecutive residues to experience shared anisotropic diffusion, the sizes of J(0) should reflect the presence of slower tumbling motions, and Q should exhibit a systematic and sequence-persistent pattern. If Q adheres closely to the isotropic expectation (Eq. [Disp-formula Ch1.E10]), it would appear that peptide plane dynamics in IDPs are isotropic and well probed by conventional ${}^{15}N$ relaxation alone. If Q and the sizes of J(0) imply systematic and correlated deviations from the isotropic case (Eq. [Disp-formula Ch1.E10]), it would hint towards different inherent mobilities in the peptide plane such as the 3D GAF-type [Bibr bib1.bibx4] dynamics predicted by [Bibr bib1.bibx55]. Indeed, the sensitivity of Q along the $C^{\alpha }C^{\alpha }$ direction illustrates the potential of $C^{\prime }/C^{\prime }C^{\alpha }$ CCR in investigating this type of motion.

Thus, while introduced and assessed in terms of diffusion anisotropy, we expect the combination of transversal and longitudinal $C^{\prime }/C^{\alpha }C^{\prime }$ CCR rates to prove informative even outside this limited scope. For the locally dominated dynamics of IDPs in particular, differences and similarities to the ${\mathrm{NH}}^{N}$ spectral density can provide valuable structural insights even without invoking specific dynamic models. As the previously highlighted studies demonstrate, MD simulations can be expected to play a key role in rationalizing possible sources of anisotropy for different protein systems ranging from “fully disordered” [Bibr bib1.bibx38] to partially structured [Bibr bib1.bibx55]. In addition, the spectral densities can also be evaluated directly. While the proposed experiments do not allow us to map $J_{C^{\prime }C^{\alpha },xx}$ and $J_{C^{\prime }C^{\alpha },yy}$ individually, the contributions of different Larmor frequencies are fully separated. Graphical representations in particular can provide model-independent intuition about the timescales at play [Bibr bib1.bibx25]. Expressions such as J(0)-J(ω) [Bibr bib1.bibx25], intended to suppress the contribution of faster timescales (cf. Fig. [Fig Ch1.F1]), are available as well. More extensive analysis could be realized using general frameworks such as correlation time distributions [Bibr bib1.bibx31] or dynamic detectors [Bibr bib1.bibx59].

## 5 Conclusions

On the occasion of Geoffrey Bodenhausen's 70th anniversary, we built on his extensive body of work to conceptualize experimental means for the investigation of anisotropic segmental dynamics in IDPs. Spectral density mapping protocols based on transversal and longitudinal CCR of ${\mathrm{NH}}^{N}$ were translated to the $C^{\alpha }C^{\prime }$ spin pair of the same peptide plane. By isolating and comparing the zero frequency contributions, we derived an intuitive experimental measure for the presence of anisotropic dynamics in IDPs. Building on the simplified image of a symmetric top, we show that pronounced anisotropic tumbling of extended chain and α -helical segments should be readily detectable. But even outside the context of this simplistic model, contributions of different frequencies can be separated and assessed similarly to spectral density mapping protocols. Interestingly, the required measurement of longitudinal $C^{\prime }/C^{\alpha }C^{\prime }$ CCR has not been investigated before. Hence, a simple proof of concept for a possible measurement scheme was provided. To further substantiate and explore the presented concepts in an experimental setting, a systematic evaluation of different pulse sequences is currently under preparation in our lab.

While detecting and quantifying the presence of anisotropy in IDP dynamics might seem like a humble academic endeavor, we believe it to be an important step not only towards a better understanding of this important protein family but also towards immediate applications in biological and biomedical research as well as drug design. We thus take particular delight from the fact that Geoffrey Bodenhausen's *l'art pour l'art* pulse sequence design is also a telling testimony for the unforeseeable impact of non-utilitarian basic research driven by and inspired by scholarly thinking.

## Supplement

10.5194/mr-2-557-2021-supplement The supplement related to this article is available online at: https://doi.org/10.5194/mr-2-557-2021-supplement.

## Data Availability

The preliminary NMR data shown for ubiquitin (Fig. 3) are available from the authors upon request.

## Code availability

The Python code used for simulations and plotting (Figs. 1 and 4) is provided as a Jupyter Notebook in the Supplement.

## Appendix

The supplement related to this article is available online at: https://doi.org/10.5194/mr-2-557-2021-supplement.
