# Supplementary material for: Detecting anisotropic segmental dynamics in disordered proteins by cross-correlated spin relaxation
Source: Magn Reson (Gott). 2021 Jul 6;2(2):557–69. doi: 10.5194/mr-2-557-2021 (PMC10539831; doi:10.5194/mr-2-557-2021)
Supplement: The supplement related to this article is available online at: https://doi.org/10.5194/mr-2-557-2021-supplement. [file mr-2-557-supplement.zip › mr-2-557-2021-supplement-title-page.pdf]

Supplement of Magn. Reson., 2, 557–569, 2021
https://doi.org/10.5194/mr-2-557-2021-supplement
© Author(s) 2021. CC BY 4.0 License.

Supplement of

Detecting anisotropic segmental dynamics in disordered proteins by cross-correlated spin relaxation

Clemens Kauffmann et al.

Correspondence to: Clemens Kauffmann (clemens.kauffmann@univie.ac.at) and Robert Konrat (robert.konrat@univie.ac.at)

- mr-2-557-2021-supplement-title-page.pdf
- code.ipynb

The copyright of individual parts of the supplement might differ from the article licence.
